# Dual subcellular compartment delivery of doxorubicin to overcome drug resistant and enhance antitumor activity

**DOI:** 10.1038/srep16125

**Published:** 2015-11-04

**Authors:** Yan-feng Song, Dao-zhou Liu, Ying Cheng, Miao Liu, Wei-liang Ye, Bang-le Zhang, Xin-you Liu, Si-yuan Zhou

**Affiliations:** 1 Department of Pharmaceutics, School of Pharmacy, Fourth Military Medical University, Xi’an, 710032, China; 2 Department of Pharmacy, Tangtu Hospital, Fourth Military Medical University, Xi’an 710032, China

## Abstract

In order to overcome drug resistant and enhance antitumor activity of DOX, a new pH-sensitive micelle (DOX/DQA-DOX@DSPE-hyd-PEG-AA) was prepared to simultaneously deliver DOX to nucleus and mitochondria. Drug released from DOX/DQA-DOX@DSPE-hyd-PEG-AA showed a pH-dependent manner. DOX/DQA-DOX@DSPE-hyd-PEG-AA induced the depolarization of mitochondria and apoptosis in MDA-MB-231/ADR cells and A549 cells, which resulted in the high cytotoxicity of DOX/DQA-DOX@DSPE-hyd-PEG-AA against MDA-MB-231/ADR cells and A549 cells. Confocal microscopy confirmed that DOX/DQA-DOX@DSPE-hyd-PEG-AA simultaneously delivered DQA-DOX and DOX to the mitochondria and nucleus of tumor cell. After DOX/DQA-DOX@DSPE-hyd-PEG-AA was injected to the tumor-bearing nude mice by the tail vein, DOX was mainly found in tumor tissue. But DOX was widely distributed in the whole body after the administration of free DOX. Compared with free DOX, the same dose of DOX/DQA-DOX@DSPE-hyd-PEG-AA significantly inhibited the growth of DOX-resistant tumor in tumor-bearing mice without obvious systemic toxicity. Therefore, dual subcellular compartment delivery of DOX greatly enhanced the antitumor activity of DOX on DOX-resistant tumor. DOX/DQA-DOX@DSPE-hyd-PEG-AA has the potential in target therapy for DOX-resistant tumor.

Doxorubicin (DOX) is one of the most commonly used broad-spectrum antitumor chemotherapeutic drugs because of its exact curative effect. For example, DOX is a first-line drug to treat breast cancer. However, long term use of DOX can induce multi-drug resistance (MDR) and serious cardiac toxicity^1^,^2^,^3^,^4^. The main target of DOX is nucleus DNA. Over-expression of p-glycoprotein (p-gp) or other drug transporter on DOX-resistant tumor cell resulted in the efflux of DOX from the tumor cells^5^, which reduce the accumulation of DOX in nucleus and decrease the antitumor efficacy of DOX, subsequently, lead to the recurrence of tumor^6^,^7^. In theory, simultaneous delivery of DOX to multi-subcellular target of DOX in tumor cell can improve the antitumor effect of DOX on drug-resistant tumor as well as on normal tumor.

In addition to nucleus, mitochondria were found to be another important target of DOX^8^,^9^,^10^,^11^,^12^. This is because that DOX can damage mitochondrial DNA (mtDNA) and induce the increase of reactive oxygen species (ROS) in mitochondria, which led to the dysfunction of mitochondria and the reduction of ATP production, subsequently, resulted in the apoptosis of tumor cell and the reduction of ATP-dependent drug efflux^13^,^14^,^15^. Thus, if DOX can be simultaneously delivered to nucleus and mitochondria of tumor cells, the antitumor efficacy of DOX will be greatly enhanced. It has been reported that delocalized lipophilic cations (DLCs) can accumulate in the cell mitochondria because of the high membrane potential of mitochondria (−150 to −180 mV)^16^,^17^. Dequalinium (DQA) is one of the delocalized lipophilic cations. DQAsomes have been investigated for their potentials in delivering mitochondrial DNA to mitochondria^18^,^19^. The results indicated that DQAsomes were able to deliver pDNA to the mitochondria without losing their pDNA load^20^. This result implied that DQA was a superior mitochondrial target ligand. Recently, dequalinium-doxorubicin conjugate (DQA-DOX) was firstly synthesized by our group, its structure is showed in Fig. 1. When DQA-DOX was cultured with DOX-resistant MCF-7/ADR cells, DQA-DOX mainly distributed in the mitochondria of MCF-7/ADR cells and showed high cytotoxicity on MCF-7/ADR cells *in vitro*^21^. However, DQA-DOX is a small molecular compound, it can’t specifically accumulate in the mitochondria of tumor cells *in vivo* after it is administered. Thus, it has very important significance to set up an active drug delivery system to deliver DOX and DQA-DOX to tumor cell *in vivo*.

Sigma receptor is over-expressed in many tumor cells such as non-small cell lung carcinoma, breast cancer, melanoma and prostate cancer^22^,^23^,^24^,^25^. The high affinity of anisamide (AA) to sigma receptor has been used in the target therapy of a variety of tumors on animal^26^,^27^,^28^,^29^,^30^,^31^.

In this study, by using AA as a tumor cell target ligand, amphiphilic pH-sensitive material DSPE-hyd-PEG-AA was synthesized. DQA-DOX and free DOX was simultaneously entrapped in DSPE-hyd-PEG-AA micelle. When DOX/DQA-DOX@DSPE-hyd-PEG-AA was uptaken by tumor cells, the micelle was disassembled in the endolysosome. Consequently, DQA-DOX and DOX diffused to the mitochondria and nucleus respectively (Fig. 2). The antitumor activity of DOX/DQA-DOX@DSPE-hyd-PEG-AA was greatly enhanced in DOX sensitive tumor cells as well as in DOX resistant tumor cells.

## Materials and Methods

### Materials

Dicyclohexylcarbodiimide (DCC), N-hydroxysuccinimide (NHS), 1-ethyl-3-(3-dimethylaminopropyl)carbodiimide (EDC), dimethylaminopyridine (DMAP), p-anisoyl chloride, 4-acetylbenzoic acid, aminocaproic acid, 1,2-distearoyl-sn-glycero-3-phosphoethanol- amine (DSPE), 5,5’,6,6’-tetrachloro-1,1’,3,3’-tetraethylbenzimidazolyl- carbocyanine iodide (JC-1) and succinic anhydride were bought from J&K CHEMICA (Beijing, China). Dequalinium, 3-(4,5-dimethylthiazol-2-yl)-2,5-diphenyltetrazolium bromide (MTT) were bought from Sigma-Aldrich company (St. Louis, MO, USA). H_2_N-PEG-NH_2_ (average molecular weight was 4000) was purchased from Shanghai Yare Biotech Inc.(Shanghai, China). Doxorubicin was supplied by Hisun Pharmaceutical Co. (Zhejiang, China). RPMI1640 medium, 4’,6-diamidino-2-phenylindole (DAPI), Lysotracker green and Mitotracker green were obtained from Invitrogen Technologies Company (Carlsbad, USA). All other chemicals were purchased from commercial supplier.

Female athymic nude mice were supplied by the Experimental Animal Center, Fourth Military Medical University. Sixteen female athymic nude mice were used in the experiment. The strain of athymic nude mice was BALB/c-nu. The age was six weeks old, and the body weight was about 21 ~ 23 g. Animal experiment was carried out according to the protocols that were approved by the Animal Care and Use Committee, Fourth Military Medical University (approval number: 15004).

### Cell lines and culture conditions

A549 cell is non-small cell lung carcinoma cell line, on which sigma receptor is high-expressed^22^,^23^. A549 cell was supplied by Institute of Biochemistry and Cell Biology, Chinese Academy of Science, Shanghai, China. MDA-MB-231 cell is human breast cancer cell, on which sigma receptor is over-expressed^22^,^24^,^27^. MDA-MB-231 cell was supplied by Institute of Biochemistry and Cell Biology, Chinese Academy of Science, Shanghai, China. MDA-MB-231/ADR cell is DOX resistant cell, which was induced by our lab. The doxorubicin resistance of MDA-MB-231 cells was induced *in vitro* by incubating MDA-MB-231 cell with increasing concentrations of doxorubicin (from 0.2 μg/ml to 2 μg/ml) for fifteen weeks^32^,^33^. The IC_50_ of doxorubicin on MDA-MB-231/ADR cells was 25 to 30-fold higher than that on MDA-MB-231 cells. Cells were cultured in RPMI 1640 with 10% fetal bovine serum, 100 u/ml penicillin, 100 mg/ml streptomycin and 5% CO_2_ at 37 °C under fully humidified conditions. The cell culture medium was changed every 24 h.

### Synthesis of DSPE-hyd-PEG-AA conjugate

The synthetic route for DSPE-hyd-PEG-AA is showed in supplementary figure 1.

### Synthesis of AA-aminocaproic acid conjugate

Aminocaproic acid (0.13 g, 1 mmol) was dissolved in 2 ml anhydrate dichloromethane. p-Anisoyl chloride (0.2 g, 1.2 mmol) was dispersed in 1 ml anhydrate dichloromethane. The p-anisoyl chloride solution was dropwise added into aminocaproic acid solution. The reaction mixture was stirred for 24 h at room temperature. The dichloromethane was removed by rotary evaporator. The residue was dissolved in ethyl acetate and was washed with 0.5 mol/l HCl. The organic phase was collected and ethyl acetate was removed by vacuum rotary evaporator. Finally, the residue was purified through silica gel column. The yield of AA-aminocaproic acid conjugate was 87%.

### Synthesis of AA-PEG-NH_2_

The AA-aminocaproic acid conjugate (5.6 mg, 0.025 mmol), DCC (5.1 mg, 0.025 mmol) and DMAP (0.3 mg, 0.025 mmol) were dissolved in 3 ml dichloromethane, and the mixture was stirred at room temperature for 5 h. Then the reaction mixture was dropwise added into the dichloromethane solution containing H_2_N-PEG-NH_2_ (100mg, 0.025 mmol). After the reaction mixture was stirred at room temperature for 24 h, N,N’-dicyclohexylurea in reaction the mixture was removed by filtration. The filtrate was collected and the dichloromethane was removed by vacuum rotary evaporator. Then the residue was dissolved in water and transferred to a dialysis bag (molecular weight cut off was 1000 Da) to dialyze in water for 2 days. After that, the reaction product was collected by lyophilization. The product was further purified by using column of Sephadex G-25^34^. The targeting component was freeze-dried to get white solid powder. The yield of AA-PEG-NH_2_ was 69%.

### Synthesis of AA-PEG-4-acetylbenzoic acid

4-Acetylbenzoicacid (13 mg, 0.08 mmol), DCC (16.4 mg, 0.08 mmol) and DMAP (0.8 mg, 0.008 mmol) were dissolved in 2 ml dichloromethane. The reaction mixture was stirred for 5 h at room temperature, then AA-PEG-NH_2_ (187 mg, 0.04 mmol) was added into the solution. After the reaction mixture was stirred at room temperature for another 12 h, the N,N’-dicyclohexylurea in reaction mixture was removed by filtration. The filtrate was collected and the dichloromethane was removed by vacuum rotary evaporator. Then the residue was dissolved in water and transferred to dialysis bag (molecular weight cut off was 1000 Da) to dialyze in water for 2 days. After that, the reaction product was collected by freeze-drying. The yield of AA-PEG-4-acetylbenzoic acid conjugate was 50%.

### Synthesis of DSPE-hemisuccinate

DSPE (50 mg, 0.06 mmol) was dissolved in 2 ml chloroform. Then succinic anhydride (14 mg, 0.14 mmol, dissolved in 0.5 ml DMSO) and 60 μl triethylamine were added into the chloroform solution. The reaction mixture was stirred for at room temperature 24 h. After that, 45 ml acetone was added. The reaction mixture was placed in −20 °C for an overnight. The crystal of DSPE-hemisuccinate was collected by filtration.

### Synthesis of DSPE-succinic hydrazide

DSPE-hemisuccinate (50 mg, 0.06 mmol), DCC (20 mg, 0.09 mmol) and DMAP (11.9 mg, 0.10 mmol) were dissolved in 2 ml chloroform, and the reaction mixture was stirred for 5 h at room temperature. Then Boc-NH-NH_2_ (18.7 mg, 0.14 mmol) and 30 μl triethylamine were added. The reaction mixture was stirred at room temperature for 24 h. The product DSPE-NH-NH_2_-Boc was purified by using silica gel column. DSPE-NH-NH_2_-Boc was dissolved into 2 ml dichloromethane, and 2 ml trifluoroacetic acid was added. The reaction mixture was stirred at room temperature for 2 h. After that, the reaction mixture was diluted by adding 10 ml water, and the product was extracted by dichloromethane. The organic phase was dried by Na_2_SO_4_, and DSPE-succinic hydrazide was collected after removing dichloromethane by using a vacuum rotary evaporator.

### Synthesis of DSPE-hyd-PEG-AA

AA-PEG-4-acetylbenzoic acid (150 mg, 0.03 mmol), DSPE-succinic hydrazide (58.7mg, 0.06 mmol) and 20 μl trifluoroacetic acid (TFA) were dissolved in 2 ml dichloromethane. The reaction mixture was stirred at room temperature for 24 h. After dichloromethane was removed by vacuum rotary evaporator, the residue was diluted with water and transferred to dialysis bag (molecular weight cut off was 1000 Da) to dialyze in water for 2 days. Finally, the product was collected by freeze-drying.

### Preparation of drug loaded micelle

The DOX and DQA-DOX loaded micelle (DOX/DQA-DOX@DSPE-hyd-PEG-AA) was prepared by emulsion-solvent evaporation method. In brief, DSPE-hyd-PEG-AA (10.0 mg) was dissolved in 5 ml dichloromethane. DOX (2.0 mg) and DQA-DOX (2.0 mg) was dissolved in 20 ml of 3.0% PVA aqueous solution. The dichloromethane solution containing DSPE-hyd-PEG-AA was dropwise added into drug-containing water phase under the condition of stirring at 4000 rpm. The resulted mixture solution was stirred at room temperature for 4 h to completely remove the dichloromethane. The micelle was collected and washed 3 times with deionized water by centrifugation at 8000 × g for 10 minutes. The micelle only entrapped DOX (DOX@DSPE-hyd-PEG-AA) was prepared with the same method.

The micelle size, zeta potential and polydispersity index were detected at 25 °C by using dynamic light scattering (DLS, Beckman Coulter Particle Analyzer, Fullerton, California, USA). All measurements were performed in triplicate. The morphology of micelles was observed by using transmission electron microscopy (TEM, JEOL-100CXII, Japan)^34^. The drug loading, encapsulation efficiency and critical micelle concentration (CMC) was detected according to the literature by using 970 CRT fluorescence spectrophotometer (Shanghai Precision and Scientific Instrument Co. Ltd, Shanghai, China)^35^,^36^. In order to study the effect of pH on the stability of micelles, the micelles were dispersed into phosphate buffer saline (PBS) at different pH (7.4 and 5.0, containing 20% fetal bovine serum), and the particle size and polydispersity index were measured at different time point.

### *In vitro* drug release

The release of drug from DOX/DQA-DOX@DSPE-hyd-PEG-AA and DOX@DSPE-hyd-PEG-AA was conducted by using the dialysis method. In short, DOX/DQA-DOX@DSPE-hyd-PEG-AA or DOX@DSPE-hyd-PEG-AA (5mg) was dispersed in 4 ml PBS at different pH (7.4, 6.5, 5.0). Then they were moved to a dialysis bag (molecular weight cut off: 1000 Da) and dialyzed against 40 ml of PBS (pH 7.4, 6.4, 5.0) at 37 °C. At predetermined time intervals, 2.0 ml sample was taken out from released medium, and 2.0 ml fresh PBS was supplemented immediately. Then the drug content in the release medium was detected by fluorescence spectrophotometer. Each study was conducted in triplicate.

### *In vitro* cytotoxicity

The cytotoxicity of free DOX, DQA-DOX, DOX@DSPE-hyd-PEG-AA and DOX/DQA-DOX@DSPE-hyd-PEG-AA were tested on MDA-MB-231/ADR cells and A549 cells by MTT method. Cells were seeded in 96-well plate (5 × 10^3^ cells per well) and incubated for 24 h. The culture medium was replaced by fresh culture medium that contained different concentration of free DOX, DQA-DOX, DOX@DSPE-hyd-PEG-AA and DOX/DQA-DOX@DSPE-hyd-PEG-AA. The equivalent DOX concentration was 1, 5, 10 and 50 μmol/l. At 48 h, 20 μl MTT (5 mg/ml) was added to the plate and incubated for 4 h. Finally, the medium of each well was replaced by 150 μl DMSO, and cell viability was detected by microplate reader (Bio-Rad Laboratories, Inc. Richmond, California, USA). In order to verify the function of anisamide (AA) in micelle, the MDA-MB-231/ADR cells were incubated with DOX/DQA-DOX@DSPE-hyd-PEG-AA (the equivalent DOX concentration was 10 μmol/l) and different concentration of anisamide for 48 h. The anisamide concentration was 0.2, 2, 10 and 50 μg/l. The cell viability was tested with the above method.

### The caspase3 activity assay

The Caspase 3 Activity Assay Kit (Beyotime Institute of Biotechnology, Jiangsu, China) was used to evaluate the effect of DOX/DQA-DOX@DSPE-hyd-PEG-AA on the activity of caspase3 in tumor cell^37^. Briefly, cells were seeded in dishes and incubated for 24 h. The culture medium was replaced by fresh culture medium that contained different concentration of DOX, DQA-DOX, DOX@DSPE-hyd-PEG-AA and DOX/DQA-DOX@DSPE-hyd-PEG-AA. The equivalent DOX concentration was 10 and 50 μmol/l. After 24 h, cells were harvested and washed with PBS. After centrifugation, the cells were collected and re-suspended in pyrolysis liquid. The cytolysis was centrifugated for 15 min at 4 °C. Finally, 40 μl of the supernatant was removed to a 96-well plate, followed by mixturing with 10 μl Ac-DEVD-pNA (2 mmol/l) and buffer. After 1 h, the absorbance at 405 nm was detected by Bio-Rad Microplate Reader.

### Mitochondrial membrane potential determination

5,5’,6,6’-tetrachloro-1,1’,3,3’-tetraethylbenzimidazolylcarbocyanine iodide (JC-1) is an specific dye of mitochondrial membrane potential. When JC-1 bond with normal mitochondria (with high mitochondrial membrane potential), it exhibits red fluorescence (590 nm). When JC-1 bond with damaged mitochondria (with low mitochondrial membrane potential), it exhibits green fluorescence (530 nm)^38^. A549 cells and MDA-MB-231/ADR cells were seeded in 6-well plates(1×10^6^ cells/well) for 12 h. DOX@DSPE-hyd-PEG-AA and DOX/DQA-DOX@DSPE-hyd-PEG-AA (the equivalent DOX concentration was 2 μmol/l) were added and incubated for 4 h, then the cell culture medium was sucked out. The cells were washed with PBS 3 times. After that, 2 ml of JC-1 work solution (2 μmol/l) was added and incubated for 20 min. Finally cells were harvested and dispersed in JC-1 buffer solution (without JC-1). The fluorescence was detected by using fluorescent spectrophotometer. Results were calculated as the ratio between red fluorescence intensity and green fluorescence intensity.

### Cellular uptake experiment

A549 cells were planted in 24-well plate that contained a cover glass (0.5 × 10^6^ cells/well) and incubated for 24 h. The culture medium was replaced with fresh culture medium containing DOX@DSPE-hyd-PEG-AA or DOX/DQA-DOX@DSPE-hyd-PEG-AA at a concentration of 2 μmol DOX. After incubation for 4 h, the culture medium was removed, and the cells were washed with PBS (pH7.4) for three times. Then the cells were cultured with 500 μl DAPI (100 μg/ml) for 15 min. After that, the cells were washed with PBS for three times. Finally, the cells were fixed by using 1.5% formaldehyde. Cover glass was placed on the glass microscope slides and observed by using a TCS SP2 confocal microscope (Leica, Germany)^39^. In addition, the MFI (mean fluorescence intensity) in the cytoplasm and nucleus was calculated in an area of 4 μM^2^ in each sample by ImageJ software.

### Subcellular distribution

MDA-MB-231/ADR cells and A549 cells were planted in 24-well plate that contained a cover glass (0.5 × 10^6^ cells/well) and incubated for 24 h. The culture medium was replaced by fresh culture medium that contained DOX@DSPE-hyd-PEG-AA or DOX/DQA-DOX@DSPE-hyd-PEG-AA at a concentration of 2 μmol DOX/l. The cells were cultured for 30 min or 4 h. After the cells were slightly washed with PBS for three times, the cells were incubated with fresh cell culture medium that contained Lysotracker green or Mitotracker green (50 nmol/l) for 0.5 h. The cells were then slightly washed with PBS for three times. After that, the cells were incubated with DAPI (10 μg/ml) for 15 min. The cells were then slightly washed with PBS for three times, fixed for 15 min by using formaldehyde and stored at 4 °C. The fluorescent images of the cells were analyzed by using a TCS SP2 confocal microscope (Leica, Germany)^39^. Furthermore, the colocalization of DOX in lysosome, mitochondria and nucleus was calculated by using ImageJ software.

### Animal experiment

Sixteen female athymic nude mice were divided into four groups. In each group, there were 4 mice. The strain of athymic nude mice was BALB/c-nu. The age was six weeks old, and the body weight was about 21 ~ 23 g. MDA-MB-231/ADR cells (1 × 10^7^ cells/0.2 ml/animal) were subcutaneously implanted in upper back in right side of female athymic nude mice. Treatment was started when the tumor volume was about 70 mm^3^. Free DOX (10 μmol/kg) or DOX/DQA-DOX@DSPE-hyd-PEG-AA (2.0, 10 μmol DOX /kg) were intravenously administered to tumor-bearing mice every 6 days (day 1, 6 and 12). Mice were observed every day. Tumor size was measured by using a caliper and calculated as the following formula: tumor volume = LW^2^/2 (W was the short diameter of tumor and L was the long diameter of tumor). In addition, DOX usually cause the injury of normal organ. Thus, at the end of the experiment, the heart, kidney, liver, spleen and lung were removed, and their sections were stained by H&E (hematoxylin and eosin) to observe tissue damage.

For drug distribution study, free DOX (10 μmol/kg) or DOX/DQA-DOX@DSPE-hyd-PEG-AA (2.0, 10 μmol DOX /kg) were intravenously administered to the tumor-bearing nude mice. Mice were sacrificed at 24 h after drug administration. The organs and tumor tissues were collected. The red DOX fluorescence in tumor tissues and organs was detected by the Caliper IVIS Lumina II *in-Vivo* image system (Caliper Life Science, USA). Besides, the fluorescence intensity in tumor tissues and organs was quantitatively analyzed by using Living Image 4.2 software. Furthermore, in order to investigate whether DOX/DQA-DOX@DSPE-hyd-PEG-AA can deliver DOX much deeper in tumor tissue, the tumor tissue was sectioned at 5 μm thickness and stained with DAPI for confocal microscopy observation.

## Results and Discussion

### DSPE-hyd-PEG-AA characterization

The infrared spectrum and ^1^H NMR spectrum of DSPE-hyd-PEG-AA are presented in supplementary figure 2 and supplementary figure 3, respectively. In infrared spectrum, peak at 1098 cm^−1^ stood for bending vibration of C-O-C etheric bond in PEG, and the peak at 771 cm^−1^ attributed to the vibration of C-H bond in benzene ring. The peak at 1634 cm^−1^ was attributed to the stretching of the C = N bond of the hydrazone linker. The peak at 1252 cm^−1^ was attributed to the stretching of C-O-C in the AA. In ^1^H NMR spectrum, the AA in DSPE-hyd-PEG-AA was verified by the signals at peak a, b, c, d, f (δ = 2.38 ppm, 8.20 ppm, 7.74 ppm, 6.19 ppm, 1.46 ppm, respective). The PEG backbone in the conjugate was verified by the signal at peak g (δ = 3.6 ppm). The DSPE backbone in the conjugate was verified by the signal at peak h (δ = 0.9 ppm).

### Characterization of micelle

**Figure 3:**
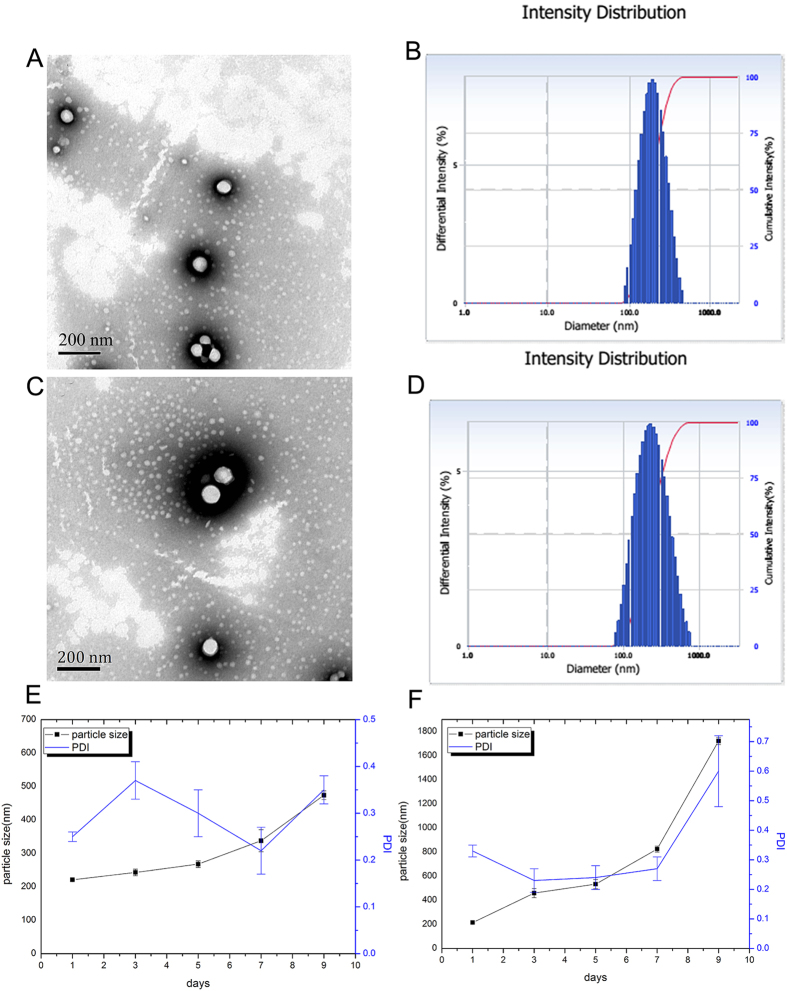
TEM image (**A**) and particle size distribution (**B**) of DOX@DSPE-hyd-PEG-AA. TEM image (**C**) and particle size distribution (**D**) of DOX/DQA-DOX@DSPE-hyd-PEG-AA. Stability of DOX/DQA-DOX@DSPE-hyd-PEG-AA in pH7.4 (**E**) and pH5.0 (**F**) PBS solution containing 20% FBS.

The drug loading, encapsulation efficiency, particle size, zeta potential, polydispersity index and critical micelle concentration (CMC) of DOX@DSPE-hyd-PEG-AA and DOX/DQA-DOX@DSPE-hyd-PEG-AA are showed in Table 1. The morphology and size distribution of DOX@DSPE-hyd-PEG-AA are showed in Fig. 3A,B, respectively. The morphology and size distribution of DOX/DQA-DOX@DSPE-hyd-PEG-AA are showed in Fig. 3C,D, respectively. Both of DOX/DQA-DOX@DSPE-hyd-PEG-AA and DOX@DSPE-hyd-PEG-AA were generally spherical in shape. The absolute value of zeta potential of DOX@DSPE-hyd-PEG-AA was bigger than that of DOX/DQA-DOX@DSPE-hyd-PEG-AA. This is probably because that DQA-DOX is a lipophilic cationic compound, which changed the surface charge of micelle. Critical micelle concentration (CMC) is an important index to evaluate the antidilution capacity of micelle. CMC value changed greatly in different micelle systems. For example, DOX-loaded mPEG-b-P(Glu-co-Phe) micelle was prepared by Lv *et al.* The CMC value of this micelle was 0.0207 mg/ml^40^. Besides, a pH sensitive (DSPE-PEG(2000)/DSPE-PEG(3400)-2C5/PHIS- PEG(2000)) micelle was prepared with CMC value of 3.6 μg/ml^41^. Recently, Li S *et al.* prepared the DOX loaded PDPA-b-PAMA micelle. The CMC value of the PDPA-b-PAMA micelle was 0.005 mg/ml^42^. The CMC value of DOX/DQA-DOX@DSPE-hyd-PEG-AA was 0.7 μg/ml. The low CMC value ensured the stability of DOX/DQA-DOX@DSPE-hyd-PEG-AA in the blood circulation even after it was significantly diluted. Thus, the low CMC value was a very important merit of DOX/DQA-DOX@DSPE-hyd-PEG-AA.

### Stability of micelle

When micelle enters into blood circulation, plasma protein such as albumin will adsorbed with micelle. The adsorption of albumin with micelle will result in the breaking of micelle structure in blood^43^. Thus, in order to investigate the stability of nanoparticle in serum, particle size is often determined in PBS containing fetal bovine serum (FBS)^44^,^45^,^46^. The stability of DOX/DQA-DOX@DSPE-hyd-PEG-AA was evaluated *in vitro* in pH7.4 PBS medium containing 20% FBS (mimicking the blood circulation environment) and pH5.0 PBS medium (mimicking the lysosome environment). The result is showed in Fig. 3E,F. The size of DOX/DQA-DOX@DSPE-hyd-PEG-AA was stable in pH7.4 medium within 5 days. However, in pH5.0 medium, the size of DOX/DQA-DOX@DSPE-hyd-PEG-AA was unstable, which was resulted from the breaking of hydrazone bond and the disassembly of micelle.

Many studies indicated that the adsorption of plasma opsonin with micelle was closely related with the surface physiochemical properties. Generally speaking, nanoparticle with high absolute value of zeta potential usually had higher opsonization rate than the same size nanoparticle with low absolute value of zeta potential^47^. Thus, nanoparticle with low absolute value of zeta potential showed low uptake by mononuclear phagocytic system (MPS), thereby displaying obvious long circulation time in blood and high accumulation in tumor tissue^48^. For example, compared with micelle with zeta potential of −26.9 mV, micelle with zeta potential of −17.5 mV showed higher tumor tissue accumulation but lower accumulation in normal organs^49^. Thus, DOX/DQA-DOX@DSPE-hyd-PEG-AA could avoid the uptake of MPS and have a long circulation time because of its relative low zeta potential.

### *In vitro* drug release studies

**Figure 4:**
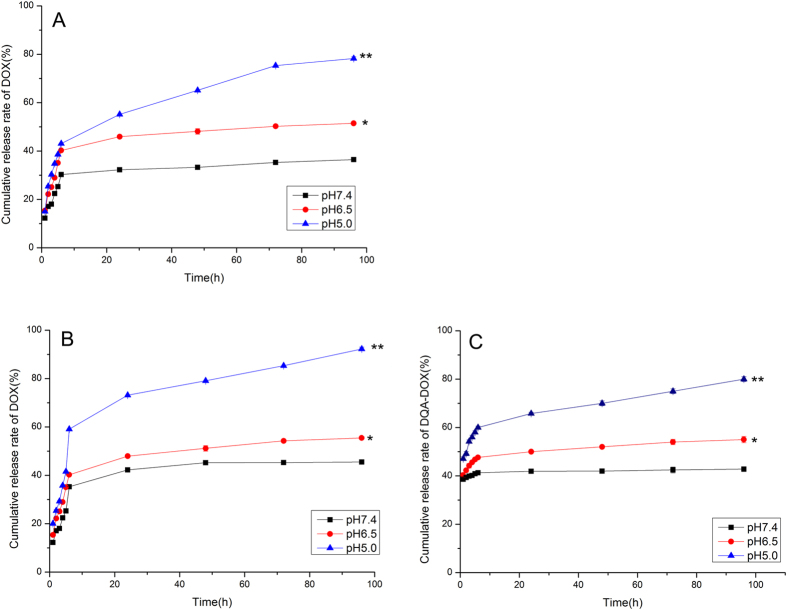
Accumulative release characteristics of DOX from DOX@DSPE-hyd-PEG-AA at different pH medium (**A**). Accumulative release characteristics of DOX (**B**) and DQA-DOX (**C**) from DOX/DQA-DOX @DSPE-hyd-PEG-AA at different pH medium. Data are presented as the mean ± SD, n = 3. *P < 0.05, **P < 0.01, vs pH7.4.

The *in vitro* drug release characteristics of DOX@DSPE-hyd-PEG-AA and DOX/DQA-DOX@DSPE-hyd-PEG-AA in pH7.4, pH6.5 and pH5.0 medium are showed in Fig. 4. The cumulative release rate of DOX and DQA-DOX from DOX/DQA-DOX@DSPE-hyd-PEG-AA showed pH-dependent manner (Fig. 4B,C). DOX/DQA-DOX@DSPE-hyd-PEG-AA released much more DOX and DQA-DOX in pH 5.0 PBS than in pH 7.4 PBS. In 8 h, more than 65% of loaded DOX and 60% of loaded DQA-DOX were released from DOX/DQA-DOX@DSPE-hyd-PEG-AA in pH 5.0 medium. While less than 35% of loaded DOX and 40% of loaded DQA-DOX were released from DOX/DQA-DOX@DSPE-hyd-PEG-AA in pH 7.4 medium in 8 h. DOX@DSPE-hyd-PEG-AA also exhibited pH-dependent drug release characteristic (Fig. 4A). In pH7.4 medium, the structure of the DOX/DQA-DOX@DSPE-hyd-PEG-AA was stable, so only a little amount of DOX was released from micelles. At pH5.0, the structure of the DOX/DQA-DOX@DSPE-hyd-PEG-AA was unstable due to the cleavage of hydrazone bond in DSPE-hyd-PEG-AA, which resulted in the disassembly of micelle, and subsequently fast release of DOX and DQA-DOX from DOX/DQA-DOX@DSPE-hyd-PEG-AA. These results implied that the DOX/DQA-DOX@DSPE-hyd-PEG-AA could effectively hinder the release of DOX and DQA-DOX from DOX/DQA-DOX@DSPE-hyd-PEG-AA in normal physiological conditions. The release of DOX and DQA-DOX from DOX/DQA-DOX@DSPE-hyd-PEG-AA was accelerated in acidic organelle, which resulted in the burst release of DOX and DQA-DOX in tumor cell, consequently enhanced the anti-tumor activity of DOX^50^,^51^,^52^. At the same time, DOX/DQA-DOX@DSPE-hyd-PEG-AA exhibited a biphasic drug release pattern as reported in many literatures^53^,^54^. In 8 h, DOX/DQA-DOX@DSPE-hyd-PEG-AA released about 60% of loaded DOX and 60% of loaded DQA-DOX in pH 5.0 PBS, respectively. In 96 h, DOX/DQA-DOX@DSPE-hyd-PEG-AA released about 95% of loaded DOX and 80% of loaded DQA-DOX in pH 5.0 PBS, respectively. The above results implied that the concentration of DOX and DQA-DOX in tumor cell could significantly increase in short time, which resulted in the saturation of p-glycoprotein and escape the efflux of DOX caused by p-glycoprotein, and large amount of DOX and DQA-DOX could traffick to nucleus and mitochondria to exert antitumor activity^55^,^56^.

### *In vitro* cytotoxicity of DOX/DQA-DOX@DSPE-hyd-PEG-AA

**Figure 5:**
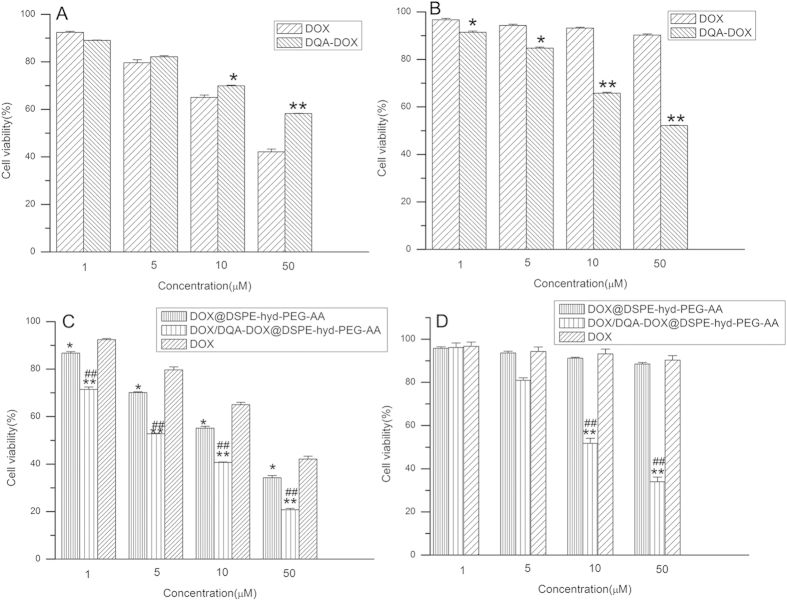
The cytotoxicity of DOX and DQA-DOX on A549 cells (**A**) and MDA-MB-231/ADR cells (**B**) in 48 h. The cytotoxicity of DOX/DQA-DOX@DSPE-hyd-PEG-AA and DOX@DSPE-hyd-PEG-AA on A549 cells (**C**) and MDA-MB-231/ADR cells (**D**) in 48 h. Data are presented as the mean ± SD, n = 3. **p < 0.01, *p < 0.05, vs the same dose of DOX. ##p < 0.01, #p < 0.05, vs the same dose of DOX@DSPE-hyd-PEG-AA.

The cytotoxicity of free DOX and free DQA-DOX on A549 cells and MDA-MB-231/ADR cells is showed in Fig. 5A,B, respectively. Compared with free DOX, free DQA-DOX exhibited lower cytotoxicity on A549 cells. However, DQA-DOX showed higher cytotoxicity on MDA-MB-231/ADR cells (DOX resistant tumor cell) as compared with free DOX. Recently, Han M *et al.* found that when DOX was conjugated with triphenylphosphonium (TPP) to form mitochondrial target conjugate TPP-DOX. TPP-DOX showed higher cytotoxicity on DOX resistant tumor cell line (MDA-MB-435/DOX cell) as compared with free DOX. But TPP-DOX exhibited lower cytotoxicity on wild type tumor cell line (MDA-MB-435 cell) as compared with free DOX. This was because TPP-DOX exhibited prior distribution to the mitochondria of the MDA-MB-435/DOX cell^57^. Accordingly, the cytotoxicity of DQA-DOX on tumor cell was similar to TPP-DOX.

The cytotoxicity of DOX/DQA-DOX@DSPE-hyd-PEG-AA and DOX@DSPE-hyd-PEG-AA on A549 cells and MDA-MB-231/ADR cells is showed in Fig. 4D, respectively. DOX/DQA-DOX@DSPE-hyd-PEG-AA and DOX@DSPE-hyd-PEG-AA showed dose-dependent cytotoxicity on MDA-MB-231/ADR cells and A549 cells. Compared with the same dose of free DOX, DOX/DQA-DOX@DSPE-hyd-PEG-AA and DOX@DSPE-hyd-PEG-AA showed greater cytotoxicity on A549 cells. Compared with the same dose of DOX@DSPE-hyd-PEG-AA, DOX/DQA-DOX@DSPE-hyd-PEG-AA exhibited higher cytotoxicity on A549 cells. In addition, the results indicated that 5% of the MDA-MB-231/ADR cells were killed by 10 μmol/l free DOX, and 7% of MDA-MB-231/ADR cells were killed by the same dose of DOX@DSPE-hyd-PEG-AA. However, the equivalent dose of DOX/DQA-DOX@DSPE-hyd-PEG-AA killed nearly 48% of MDA-MB-231/ADR cells. These results indicated that DOX/DQA-DOX@DSPE-hyd-PEG-AA exhibited high cytotoxicity both on DOX-sensitive tumor cells and DOX-resistant tumor cells. Furthermore, the above data implied that simultaneous delivery of DOX to nucleus and mitochondria greatly enhanced the antitumor activity of DOX.

**Figure 6:**
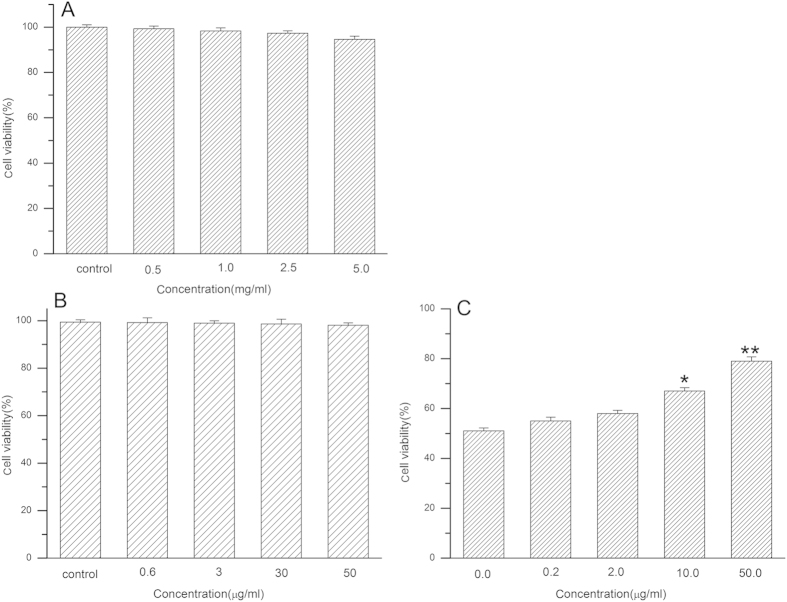
The cytotoxicity of DSPE-hyd-PEG-AA (**A**) and anisamide (**B**) on MDA-MB-231/ADR cells in 48 h. The effect of exogenous AA on the cytotoxicity of DOX/DQA-DOX@DSPE-hyd-PEG-AA on MDA-MB-231/ADR cells in 48 h (**C**). Data are presented as mean ± SD, n = 3. **p < 0.01, *p < 0.05, vs control.

The cytotoxicity of DSPE-hyd-PEG-AA on MDA-MB-231/ADR cells is showed in Fig. 6A. It was indicated that DSPE-hyd-PEG-AA had none of the cytotoxicity on MDA-MB-231/ADR cells. The effect of anisamide (AA) on the MDA-MB-231/ADR cells viability was investigated, and the result is showed in Fig. 6B. The result indicated that AA had none effect on cell viability when the concentration of AA ranged from 0.2 to 50 μg/ml. The above results implied that the cytotoxicity of DOX/DQA-DOX@DSPE-hyd-PEG-AA and DOX@DSPE-hyd-PEG-AA was caused by active component (DOX and DQA-DOX) in the micelles. Moreover, as showed in Fig. 6C, when a series concentration of exogenous AA and DOX/DQA-DOX@DSPE-hyd-PEG-AA (10 μmol/l) were co-cultured with MDA-MB-231/ADR cells, the viability of cells was increased in dose dependent manner. The decrease of cytotoxicity of DOX/DQA-DOX@DSPE-hyd-PEG-AA in the present of AA was due to the competitive inhibition effect of AA on the uptake of drug loaded micelle. This result implied that AA played an important role in the cytotoxicity of DOX/DQA-DOX@DSPE-hyd-PEG-AA.

### The caspase3 activity

**Figure 7:**
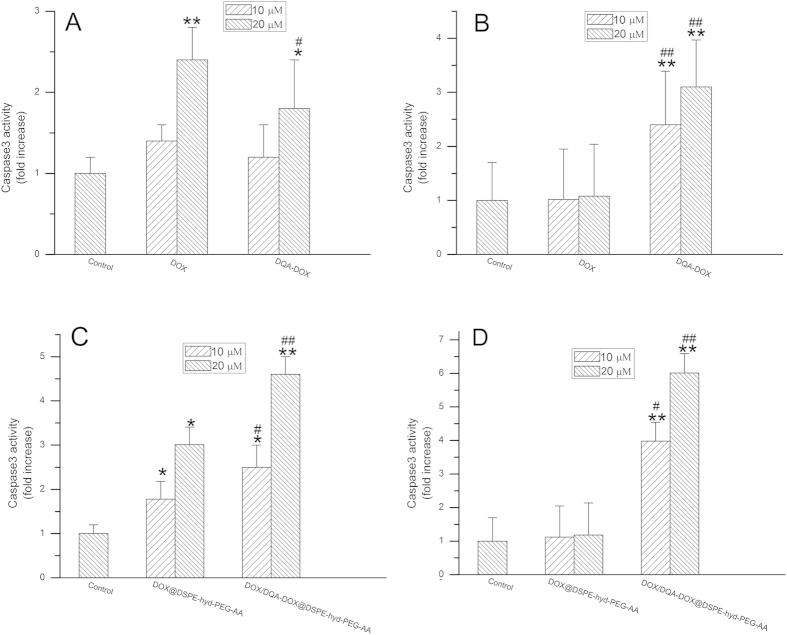
The effect of DOX and DQA-DOX on caspase3 activity in A549 cells (**A**) and MDA-MB-231/ADR cells (**B**) in 24 h. The effect of DOX/DQA-DOX@DSPE-hyd-PEG-AA and DOX@DSPE-hyd-PEG-AA on caspase3 activity in A549 cells (**C**) and MDA-MB-231/ADR cells (**D**) in 24 h. Data are presented as mean ± SD, n = 3. **p < 0.01, *p < 0.05, vs control; ##p < 0.01, #p < 0.05, vs the same dose of DOX or DOX@DSPE-hyd-PEG-AA.

A number of findings supported that cytotoxicity of DOX was resulted from inducing apoptosis^58^,^59^. Caspase3 is a key executive molecule of apoptosis. In addition, it was reported when mitochondrial membrane potential were damaged, it led to the increase of caspase3 in the cell^60^. Thus, the effects of free DOX, DQA-DOX, DOX@DSPE-hyd-PEG-AA and DOX/DQA-DOX@DSPE-hyd-PEG-AA on the level of caspase3 in tumor cell were investigated. After A549 cells and MDA-MB-231/ADR cells were treated with different concentration of free DQA-DOX for 24 h, the cell apoptosis are showed in Fig. 7A,B, respectively. Compared with free DOX, free DQA-DOX induced less apoptosis on A549 cells. But free DQA-DOX induced much more apoptosis on MDA-MB-231/ADR cells as compared with free DOX. The above results were consistent with the cytotoxicity of free DQA-DOX on A549 cells and MDA-MB-231/ADR cells.

After A549 cells and MDA-MB-231/ADR cells were treated with different concentration of DOX/DQA-DOX@DSPE-hyd-PEG-AA for 24 h, the cell apoptosis are showed in Fig. 7C,D, respectively. Compared with DOX@DSPE-hyd-PEG-AA, DOX/DQA-DOX@DSPE-hyd-PEG-AA induced much more apoptosis in A549 cells in a dose-dependent manner. Additionally, DOX@DSPE-hyd-PEG-AA could not induce significant apoptosis on MDA-MB-231/ADR cells in 24 h. But DOX/DQA-DOX@DSPE-hyd-PEG-AA induced obvious apoptosis in MDA-MB-231/ADR cells in a dose-dependent manner in 24 h. The above results implied that simultaneous delivery of DOX to nucleus and mitochondria greatly enhanced the apoptosis induce capacity of DOX. In addition, the apoptosis induce capacity of DOX@DSPE-hyd-PEG-AA and DOX/DQA-DOX@DSPE-hyd-PEG-AA was well consistent with their cytotoxicity on MDA-MB-231/ADR cells and A549 cells.

### The effect of DOX/DQA-DOX@DSPE-hyd-PEG-AA on mitochondrial membrane potential

**Figure 8:**
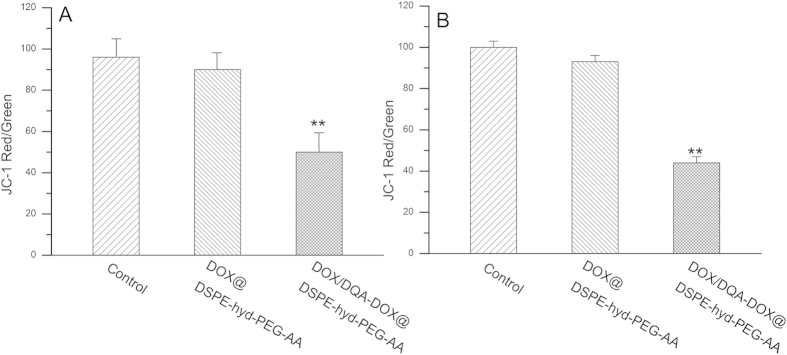
The effect of DOX/DQA-DOX@DSPE-hyd-PEG-AA and DOX@DSPE-hyd-PEG-AA on mitochondrial membrane potential of A549 cells (**A**) and MDA-MB-231/ADR cells (**B**).The equivalent DOX concentration was 2 μmol/l. Data are presented as mean ± SD, n = 3. **p < 0.01, vs the same dose of DOX@DSPE-hyd-PEG-AA.

The effect of DOX/DQA-DOX@DSPE-hyd-PEG-AA on mitochondrial membrane potential of tumor cell was detected by using JC-1 staining. The decrease of red/green fluorescence intensity indicates depolarization of mitochondria. As showed in Fig. 8A, the red/green values did not significantly decrease after A549 cells were cultured with DOX@DSPE-hyd-PEG-AA. But the red/green values significantly decreased after A549 cells were cultured with DOX/DQA-DOX@DSPE-hyd-PEG-AA. The similar results were got on MDA-MB-231/ADR cells. As showed in Fig. 8B, the red/green values did not significantly decrease after MDA-MB-231/ADR cells were cultured with DOX@DSPE-hyd-PEG-AA. But the red/green values significantly decreased after MDA-MB-231/ADR cells were incubated with DOX/DQA-DOX@DSPE-hyd-PEG-AA. The above results implied that delivery of DOX to mitochondria led to the decrease the mitochondrial membrane potential. The attenuation of mitochondrial membrane potential usually accompanied with the release of cytochrome c (Cyt-c) and the activation of caspase-9 and caspase-3. Thus, delivery of DOX to mitochondria could accelerate the apoptosis of tumor cell. In addition, one of the most important roles of mitochondria is to produce ATP. A high mitochondrial membrane potential is essential for the production of ATP. P-gp is an ATP-dependent drug efflux pump^15^,^61^. Therefore, the decrease of mitochondrial membrane potential resulted in the reduction of drug efflux mediated by P-gp and the increase of the accumulation of antitumor drug in the drug resistant-tumor cells, subsequently enhance the antitumor activity on drug-resistant tumor cells.

Generally, DOX shows a high affinity to DNA and RNA^62^. However, DNA intercalation is not the only mechanism of the antitumor activity of DOX. The multiple interactions can occur between mitochondria and DOX, which induce the mitochondrial dysfunction. Four factors are mainly responsible for mitochondrial dysfunction: (1) The high affinity of DOX to lipid bilayer leads to the disruption of mitochondrial membranes; (2) DOX can cause the release of some mitochondrial enzymes such as Cyt-c probably through the disruption of membrane, which led to a cascade effect of cell apoptosis; (3) DOX can directly inhibit mitochondrial enzymes such as cytochrome c oxidase; (4) DOX can induce the generation of free radicals in mitochondrial, which leads to calcium release, lipid peroxidation and oxidative stress^63^.

### Cellular uptake of micelles

**Figure 9:**
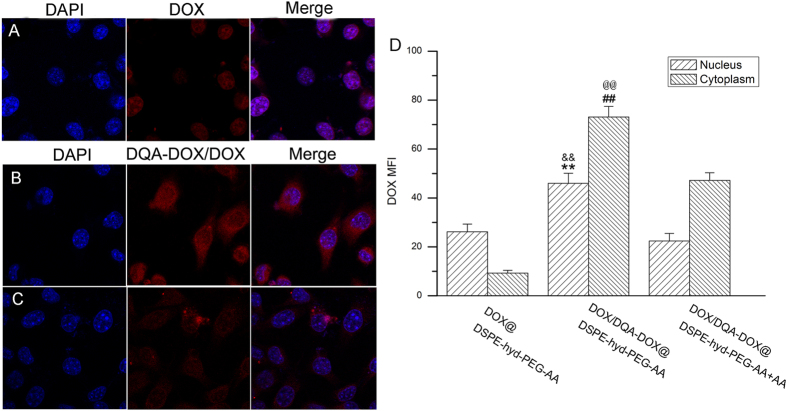
The cellular uptake of DOX@DSPE-hyd-PEG-AA (**A**), DOX/DQA-DOX@DSPE-hyd-PEG-AA (**B**) and DOX/DQA-DOX@DSPE-hyd-PEG-AA+AA (**C**) on A549 cell in 4 h. 60×oil immersion objective and 10×ocular lens. The quantitative analysis of DOX distribution in nucleus and cytoplasm on A549 cell (**D**). The pink region indicates the localization of DOX (red) in the nucleus (blue). **p < 0.01, vs nucleus of DOX/DQA-DOX@DSPE-hyd-PEG-AA+AA; &&p < 0.01, vs nucleus of DOX@DSPE-hyd-PEG-AA; ##p < 0.01 vs cytoplasm of DOX/DQA-DOX@DSPE-hyd-PEG-AA+AA; @@p < 0.01 vs cytoplasm of DOX@DSPE-hyd-PEG-AA.

The cellular uptake of DOX@DSPE-hyd-PEG-AA and DOX/DQA-DOX @DSPE-hyd-PEG-AA were evaluated by CLSM, the results are showed in Fig 9. When A549 cells were cultured with DOX@DSPE-hyd-PEG-AA, the red DOX fluorescence was mainly distributed in the nucleus. When A549 cells were cultured with DOX/DQA-DOX@DSPE-hyd-PEG-AA, large amount of red DOX fluorescence was found in the cytoplasm and nucleus. Compared with nucleus, the mean fluorescence intensity in cytoplasm was higher. This was due to the disassembly of DOX/DQA-DOX@DSPE-hyd-PEG-AA in endolysosomes, subsequently led to the burst release of DOX and DQA-DOX. Finally, DOX and DQA-DOX trafficked to the nucleus and mitochondria. When DOX/DQA-DOX@DSPE-hyd-PEG-AA and exogenous free AA were co-cultured with A549 cells, the mean fluorescence intensity in the cytoplasm and nucleus was significantly reduced. Moreover, MTT experiment indicated exogenous AA could reduce the cytotoxicity of DOX/DQA-DOX@DSPE-hyd-PEG-AA on tumor cells. These results indicated that the cellular uptake of DOX/DQA-DOX@DSPE-hyd-PEG-AA was mediated by sigma receptor.

### The sub-cellular distribution of DOX

**Figure 10:**
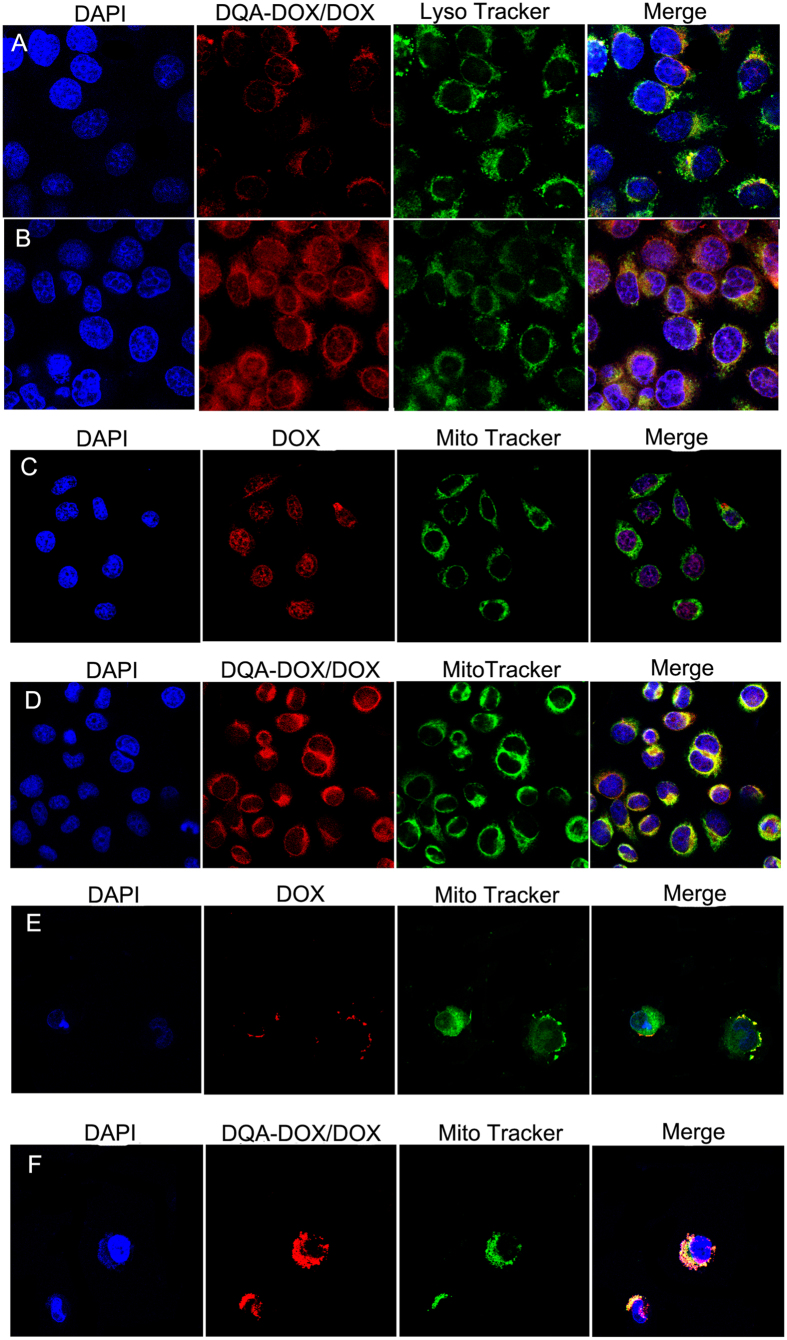
The distribution of DOX in lysosome after A549 cells were incubated with DOX/DQA-DOX@DSPE-hyd-PEG-AA at 37 °C for 30 min (**A**) and 4 h (**B**). The distribution of DOX in mitochondria after A549 cells were treated with DOX@DSPE-hyd-PEG-AA (**C**) and DOX/DQA-DOX@DSPE-hyd-PEG-AA (D) at 37 °C for 4 h. The distribution of DOX in mitochondria after MDA-MB-231/ADR cells were incubated with DOX@DSPE-hyd-PEG-AA (E) DOX/DQA-DOX@DSPE-hyd-PEG-AA (**F**) at 37 °C for 4 h. 60×oil immersion objective and 10×ocular lens. The DOX concentration was 2 μmol/l. The yellow color indicates the localization of DOX (red) in mitochondria (green) or lysosome (green). The pink region indicates the localization of DOX (red) in the nucleus (blue).

**Figure 11:**
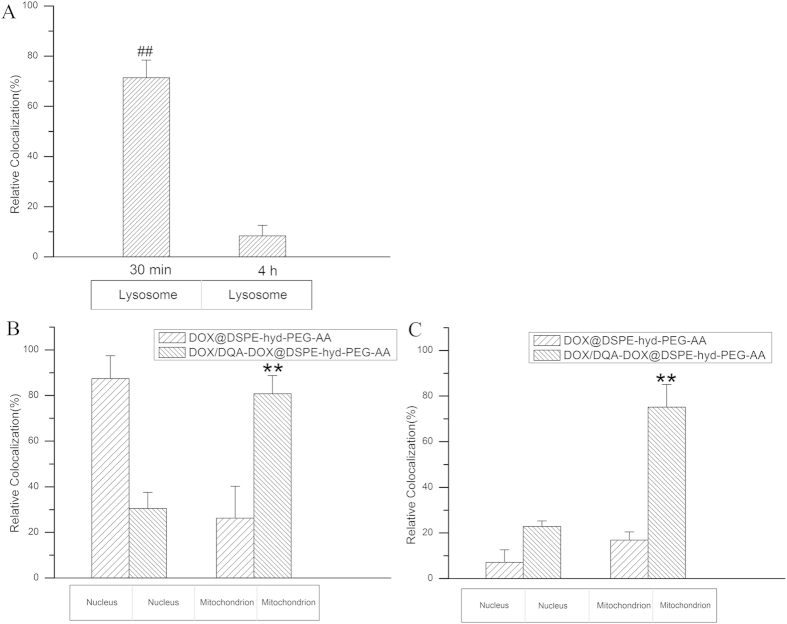
The quantitative analysis of DOX distribution in lysosome after A549 cells were incubated with DOX/DQA-DOX@DSPE-hyd-PEG-AA at 37 °C for 30 min and 4 h (**A**).The quantitative analysis of DOX distribution in nucleus and mitochondria after DOX/DQA-DOX@DSPE-hyd-PEG-AA cultured with A549 cells (**B**) and MDA-MB-231/ADR cells (**C**) at 37 °C for 4 h. **p < 0.01, vs mitochondria of DOX@DSPE-hyd-PEG-AA; ##p < 0.01, vs 4 h.

Nucleus and mitochondria are the targets of DOX. Thus, the distribution of DOX in nucleus, mitochondria and lysosome were investigated, and the results are showed in Figs 10,11. When DOX/DQA-DOX@DSPE-hyd-PEG-AA was cultured with A549 cells for 30 min, red DOX fluorescence was localized in lysosomes. But red DOX fluorescence was mainly localized in cytoplasm and nucleus after 4 h incubation. This result indicated that DQA-DOX and DOX escaped from lysosome and trafficked to cytoplasm and nucleus in 4 h. In addition, large amount of red DOX fluorescence was found in nucleus and mitochondria after DOX/DQA-DOX@DSPE-hyd-PEG-AA was cultured with A549 cells for 4 h. The above results demonstrated that DOX/DQA-DOX@DSPE-hyd-PEG-AA could deliver DOX and DQA-DOX to nucleus and mitochondria respectively, which resulted in the greater apoptosis induce capacity and higher cytotoxicity of DOX/DQA-DOX@DSPE-hyd-PEG-AA on A549 cells as compared with DOX@DSPE-hyd-PEG-AA.

When DOX@DSPE-hyd-PEG-AA was incubated with MDA-MB-231/ADR cells for 4 h, little amount of red DOX fluorescence was distributed in nucleus and mitochondria, which resulted in the low cytotoxicity of DOX@DSPE-hyd-PEG-AA on MDA-MB-231/ADR cells. When DOX/DQA-DOX@DSPE-hyd-PEG-AA was incubated with MDA-MB-231/ADR cells for 4 h, large amount of red DOX fluorescence was found in mitochondria, which resulted in the great apoptosis induce capacity and high cytotoxicity of DOX/DQA-DOX@DSPE-hyd-PEG-AA on MDA-MB-231/ADR cells.

### *In vivo* antitumor activity of DOX/DQA-DOX@DSPE-hyd-PEG-AA

**Figure 12:**
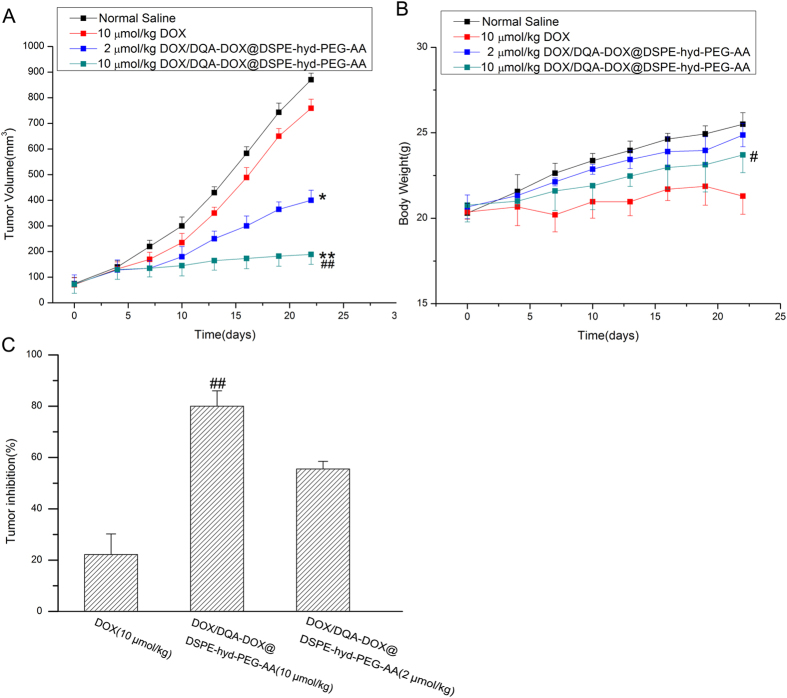
*In vivo* antitumor activity of DOX/DQA-DOX@DSPE-hyd-PEG-AA. A-the variation profiles of tumor volumes. B-body weights of tumor-bearing mice. C-the tumor inhibition of free DOX and DOX/DQA-DOX@DSPE-hyd-PEG-AA. Athymic nude mice xenografted with MDA-MB-231/ADR cells were treated with different doses of DOX/DQA-DOX@DSPE-hyd-PEG-AA (2.0, 10 μmol/kg DOX) and free DOX (10 μmol/kg) every 6 days (day 1, 6 and 12). Data are presented as mean ± SD, n = 4. **p < 0.01, *p < 0.05, vs normal saline; ##p < 0.01, #p < 0.05, vs free DOX.

**Figure 13:**
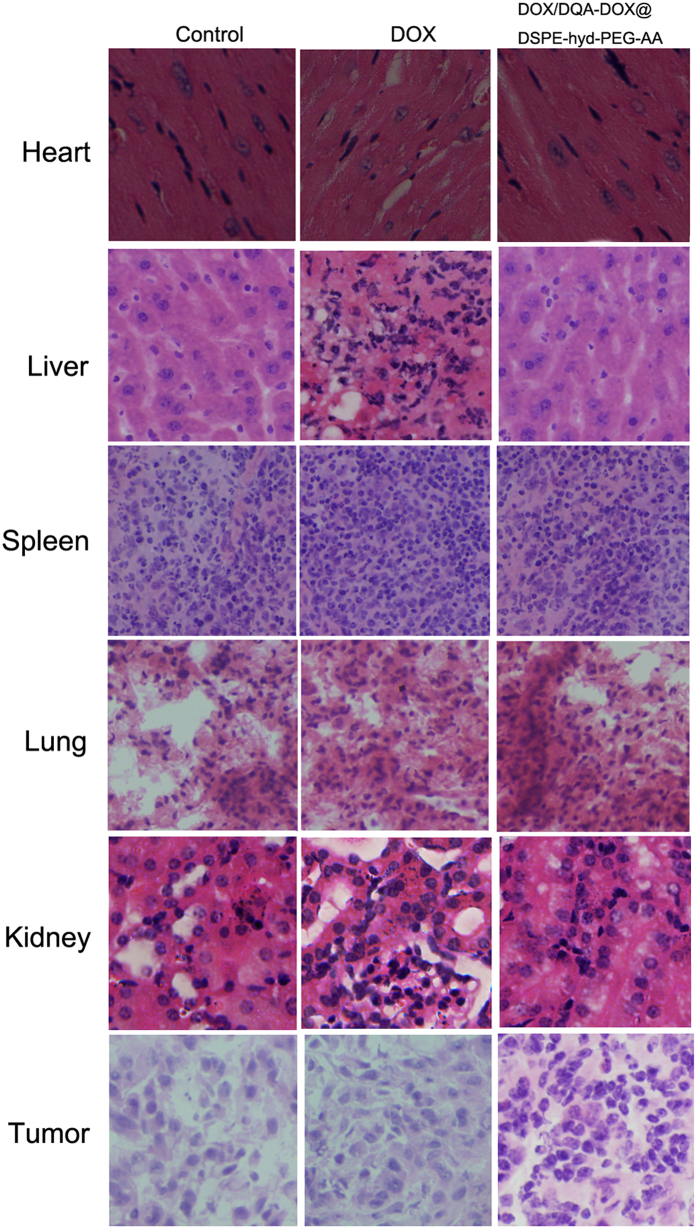
The typical H&E staining slices of normal organs and tumor tissue from tumor-bearing nude mice treated with free DOX (10 μmol DOX/kg) and DOX/DQA-DOX@DSPE-hyd-PEG-AA (10 μmol DOX/kg). 40×oil immersion objective and 10×ocular lens.

The *in vivo* antitumor activities of DOX/DQA-DOX@DSPE-hyd-PEG-AA are showed in Fig. 12. The tumor volume rapidly increased in normal saline treated and free DOX treated mice. However, compared with DOX treated group, same dose of DOX/DQA-DOX@DSPE-hyd-PEG-AA markedly delayed the tumor growth in dose-dependent manner. Besides, the tumor inhibition rate of DOX/DQA-DOX@DSPE-hyd-PEG-AA was higher than that of same dose of free DOX. Furthermore, histopathological analysis of tumor tissue was carried out to further evaluate the antitumor effect, and the representative H&E staining sections are showed in Fig. 13. Compared with tumor section from normal saline and free DOX treated nude mice, tumor section from DOX/DQA-DOX@DSPE-hyd-PEG-AA treated nude mice showed obvious vacuolation, inflammatory cell infiltration and nucleus lysis. This result indicated that DOX/DQA-DOX@DSPE-hyd-PEG-AA enhanced the toxicity of DOX to tumor tissue. The above data implied that simultaneous delivery of DOX to nucleus and mitochondria significantly increased the *in vivo* antitumor activity of DOX on DOX-resistant tumor.

### Drug distribution

**Figure 14:**
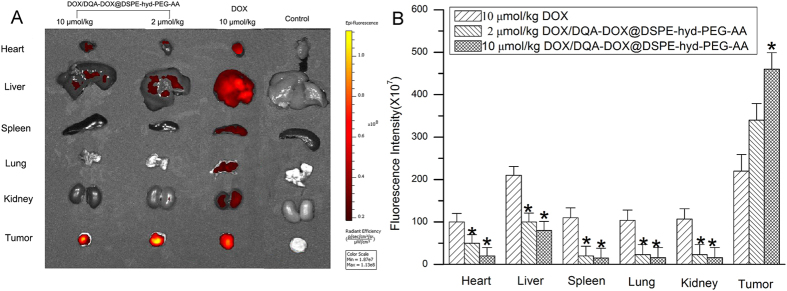
The DOX distribution in the normal organs and tumor tissue of tumor-bearing nude mice at 24 h after the injection of free DOX or DOX/DQA-DOX@DSPE-hyd-PEG-AA (**A**) by tail vein. Quantitative analysis of DOX distribution in different tissues in tumor-bearing nude mice (B). Data are presented as mean ± SD, n = 4. *p < 0.05, vs DOX treated group.

**Figure 15:**
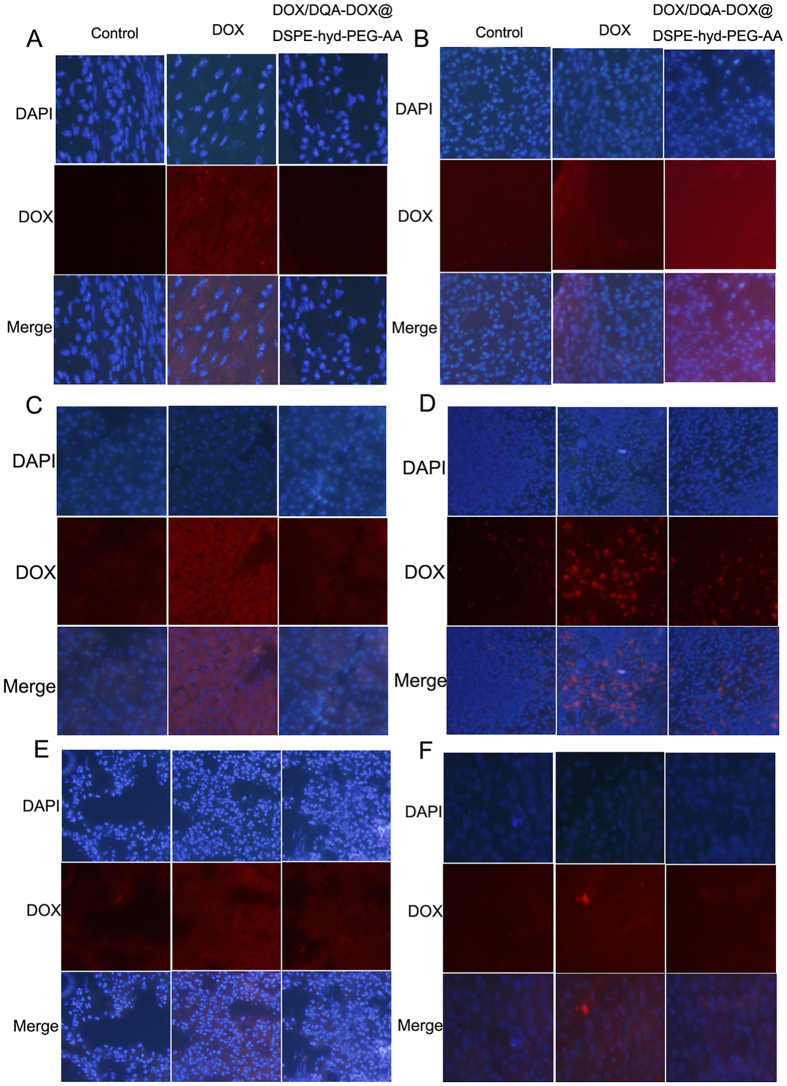
DOX distribution in section of heart (**A**), tumor (**B**), liver (**C**), spleen (**D**), lung (**E**) and kidney (**F**) detected by confocal laser scanning microscopy at 24 h after free DOX (10 μmol DOX /kg) and DOX/DQA-DOX@DSPE-hyd-PEG-AA (10 μmol DOX/kg) were injected to tumor-bearing nude mice by tail vein. 60×oil immersion objective and 10×ocular lens.

After DOX/DQA-DOX@DSPE-hyd-PEG-AA was injecteded, the distribution of DOX in tumor-bearing nude mice is showed in Fig. 14. The red DOX fluorescence was typically found in heart, lung, liver, kidney, spleen and tumor tissue after free DOX was intravenously administered. On the other hand, after DOX/DQA-DOX@DSPE-hyd-PEG-AA was intravenously administered, the red DOX fluorescence was mainly accumulated in the tumor tissue, and little amount of the red DOX fluorescence was found in heart, liver, spleen, lung and kidney. In addition, the distribution of DOX was further semi-quantitative analysis. The results are showed in Fig. 14B. Compared with free DOX treatment group, the mean fluorescence intensity was markedly higher in tumor tissues, and the mean fluorescence intensity was significantly lower in normal organs after DOX/DQA-DOX@DSPE-hyd-PEG-AA was injected via tail vein to tumor-bearing nude mice. Furthermore, the DOX distribution in section of tumor tissue and normal organ was observed by confocal microscopy, and results are showed in Fig. 15. Compared with free DOX treated tumor section, DOX/DQA-DOX@DSPE-hyd-PEG-AA treated tumor section showed more extensive distribution of red DOX fluorescence throughout the whole tumor tissue section. This result indicated that DOX/DQA-DOX@DSPE-hyd-PEG-AA could deliver DOX much deeper in tumor tissue than free DOX. Compared with normal organ section from DOX/DQA-DOX@DSPE-hyd-PEG-AA treated mice, distribution of red DOX fluorescence in normal organ section from DOX treated mice showed much extensive. These results were consistent with the result of in-Vivo Image experiment. The above results indicated that DOX/DQA-DOX@DSPE-hyd-PEG-AA mainly delivered DOX to tumor tissue and obviously reduced the accumulation of DOX in normal tissue.

### The toxicity evaluation of DOX/DQA-DOX@DSPE-hyd-PEG-AA treatment

The tumor-bearing nude mice treated with DOX/DQA-DOX@DSPE-hyd-PEG-AA showed a vigorous and healthy appearance throughout the whole experiment. Free DOX treated tumor-bearing nude mice exhibited a weakened vitality. Body weight is an indicator of systemic toxicity. The body weight of DOX/DQA-DOX@DSPE-hyd-PEG-AA treated mice increased gradually as normal saline treated mice did. Compared with DOX/DQA-DOX@DSPE-hyd-PEG-AA treated mice, body weight of DOX treated mice increased much slower (Fig. 12B). The above results indicated that DOX/DQA-DOX@DSPE-hyd-PEG-AA did not exhibit obvious systemic toxicity.

The typical H&E staining slices of heart, kidney, liver, spleen and lung of tumor-bearing nude mice that treated with normal saline, free DOX and DOX/DQA-DOX@DSPE-hyd-PEG-AA are showed in Fig. 13. No obvious histopathological changes were observed in heart, kidney, liver, spleen and lung section from DOX/DQA-DOX@DSPE-hyd-PEG-AA treated tumor-bearing nude mice. This is because that DOX/DQA-DOX@DSPE-hyd-PEG-AA delivered more DOX to tumor tissues, and reduced the accumulation of DOX in heart, kidney, liver, spleen and lung. Cardiac section from DOX treated tumor-bearing nude mice showed marked histopathological changes including neutrophils infiltration and cardiomyocyte hypertrophy. This result indicated that free DOX caused evident cardiac toxicity. This is because consider amount of DOX was accumulated in heart tissue after DOX was administered. The above results indicated that DOX/DQA-DOX@DSPE-hyd-PEG-AA significantly attenuated the toxicity of DOX to normal organ.

## Conclussion

DOX/DQA-DOX@DSPE-hyd-PEG-AA released DOX and DQA-DOX in a pH-dependent manner. DOX/DQA-DOX@DSPE-hyd-PEG-AA delivered DOX and DQA-DOX to nucleus and mitochondria simultaneously, which led to the dysfunction of mitochondria. Consequently DOX/DQA-DOX@DSPE-hyd-PEG-AA induced more apoptosis on tumor cells. DOX/DQA-DOX@DSPE-hyd-PEG-AA mainly delivered DOX to tumor tissue and obviously reduced the accumulation of DOX in normal tissue. Thus, the *in vivo* anti-tumor activity of DOX/DQA-DOX@DSPE-hyd-PEG-AA on DOX-resistant tumor was greatly enhanced. In a word, dual subcellular compartment delivery of DOX markedly increased the antitumor activity of DOX for DOX-resistant tumor. DOX/DQA-DOX@DSPE-hyd-PEG-AA has the potential in target therapy for DOX-resistant tumor.

## Additional Information

**How to cite this article**: Song, Y.-F. *et al.* Dual subcellular compartment delivery of doxorubicin to overcome drug resistant and enhance antitumor activity. *Sci. Rep.* **5**, 16125; doi: 10.1038/srep16125 (2015).

## Supplementary Material

Supplementary Information

## Figures and Tables

**Figure 1:**
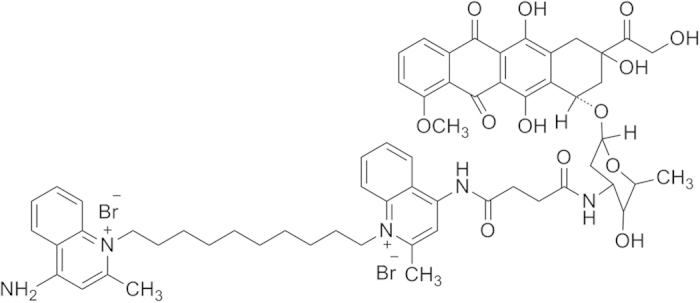
Chemical structure of DQA-DOX.

**Figure 2:**
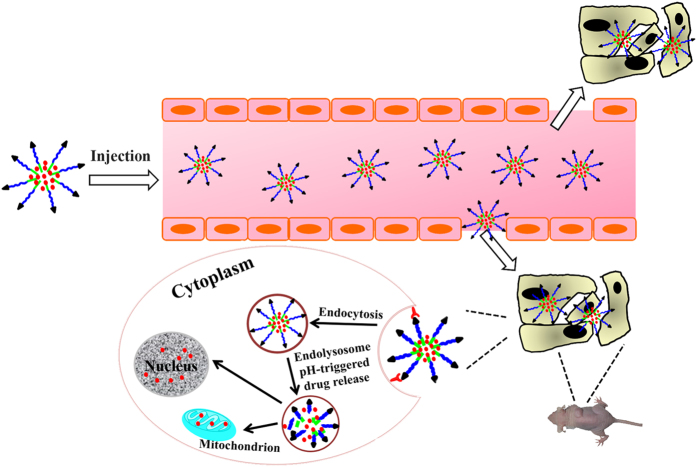
Schematic representation of pH responsive DOX/DQA-DOX@DSPE-hyd-PEG-AA for intracellular doxorubicin delivery in breast cancer. This figure was drawn by Yan-feng Song. The mouse picture was taken during the animal experiment by Yan-feng Song.

**Table 1:** Characteristics of DOX loaded micelles.

DOX loaded micelles	Particle size (nm)	Zeta potential (mV)	PDI	Drug loading (%)	Encapsulation efficiency (%)	CMC (μg/ml)
				DOX DQA-DOX	DOX DQA-DOX	
DOX@DSPE-hyd-PEG-AA	195 ± 10	−20 ± 3	0.21 ± 0.02	7.5 ± 0.6/	78.7 ± 0.2/	0.72 ± 0.17
DOX/DQA-DOX@DSPE-hyd-PEG-AA	224 ± 7	−11 ± 2	0.14 ± 0.05	6.2 ± 1.3 6.4 ± 1.5	65.7 ± 0.9 60.3 ± 1.2	0.70 ± 0.18

/-no applicable.
