# Supplementary material for: Dual subcellular compartment delivery of doxorubicin to overcome drug resistant and enhance antitumor activity
Source: Sci Rep. 2015 Nov 4;5:16125. doi: 10.1038/srep16125 (PMC4632084; doi:10.1038/srep16125)
Supplement: Supplementary Information [file srep16125-s1.pdf]

Dual subcellular compartment delivery of doxorubicin to overcome drug resistant and enhance antitumor activity

Yan-feng Song[1], Dao-zhou Liu[1#], Ying Cheng[1], Miao Liu[1],

Wei-liang Ye[1], Bang-le Zhang[1], Xin-you Liu[2]*, Si-yuan Zhou[1]*

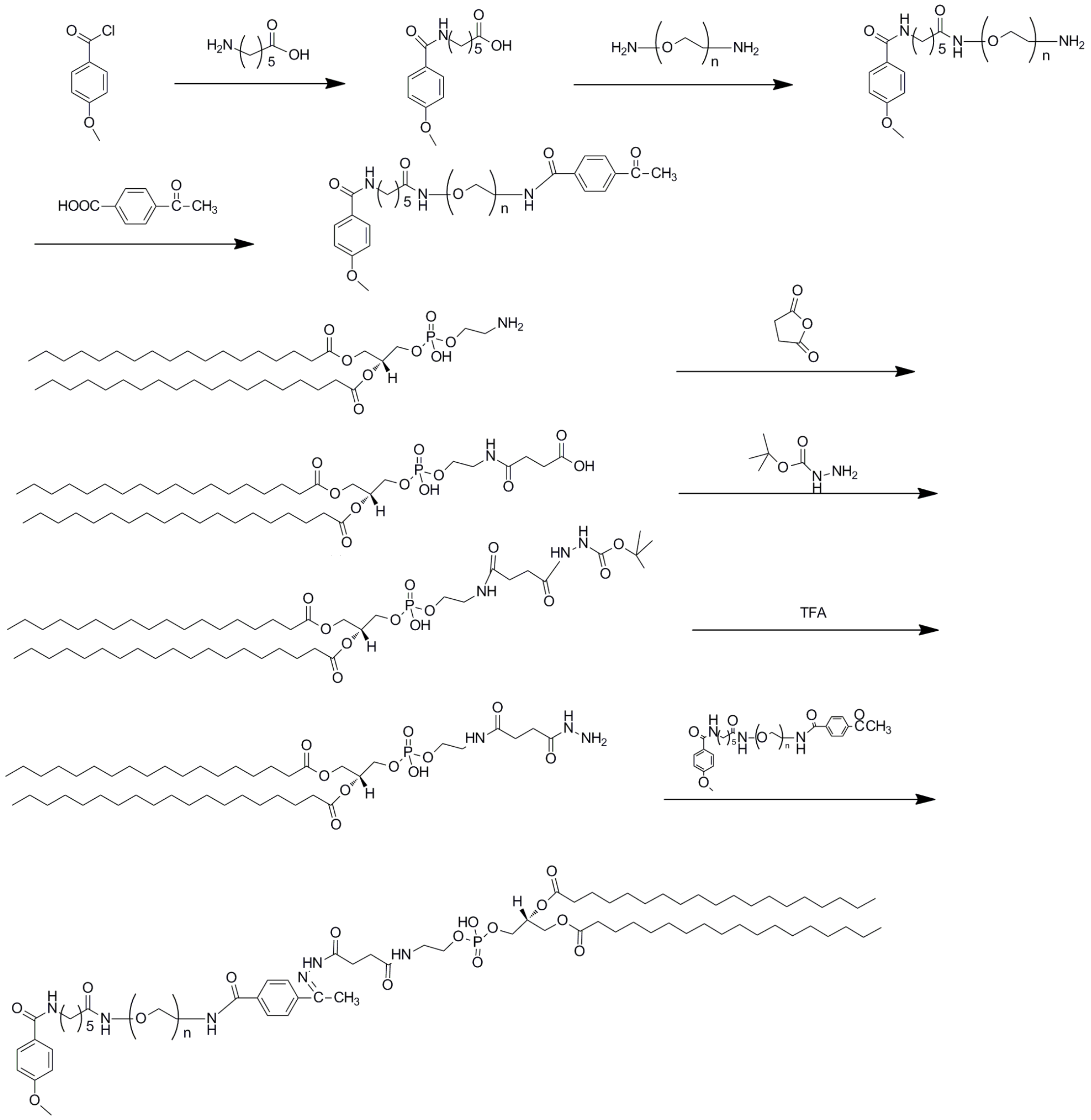

Supplementary figure 1 Synthetic scheme of DSPE-hyd-PEG-AA.

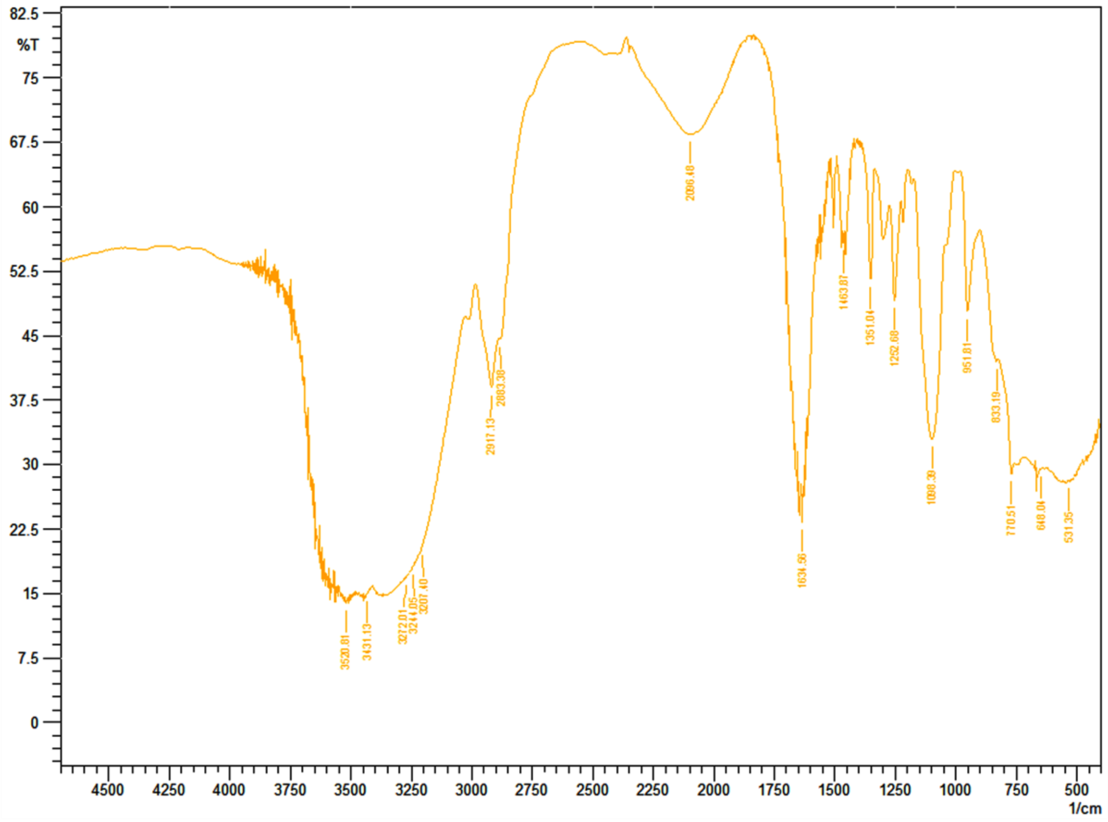

Supplementary figure2 The FTIR spectrum of the DSPE-hyd-PEG-AA.

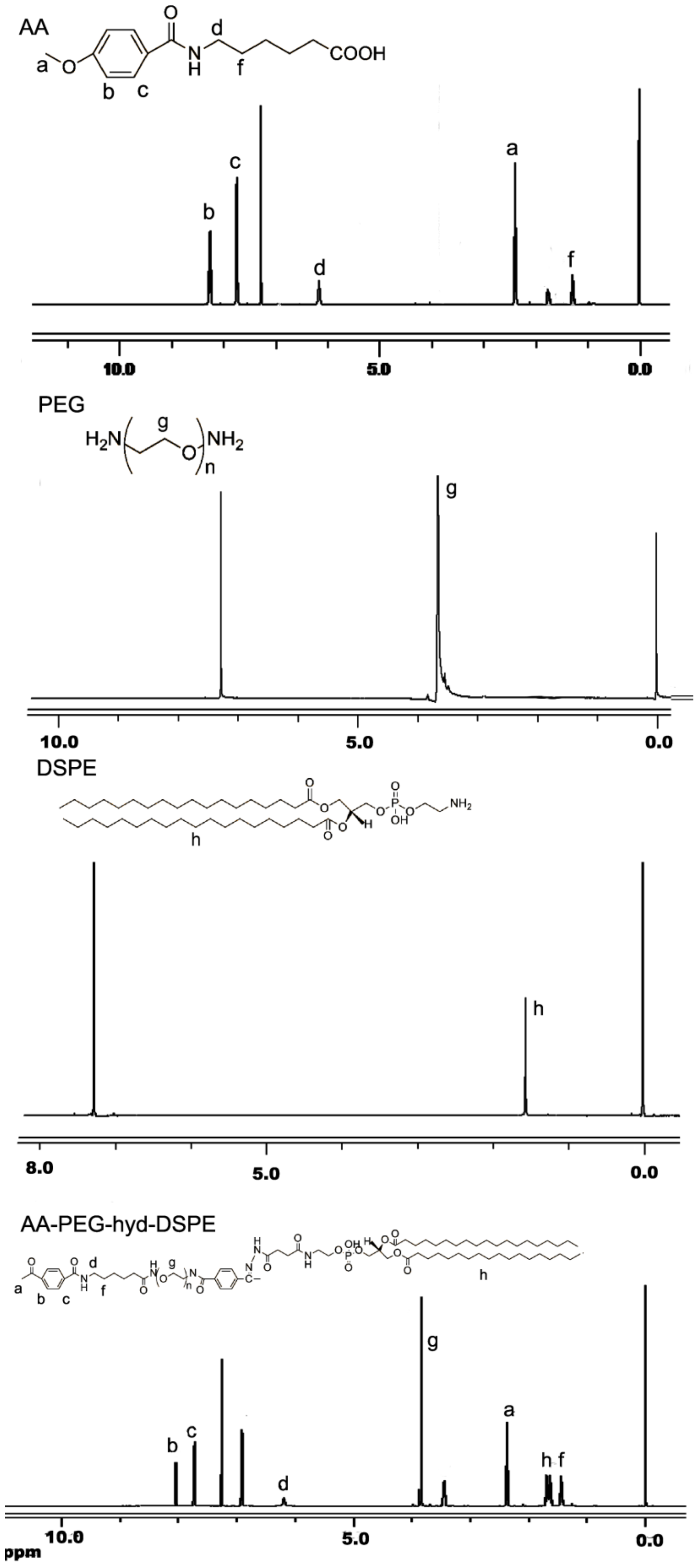
AA
COOH
a
b
c
d
f
10.0
5.0
0.0
PEG
H2N
NH2
n
g
10.0
5.0
0.0
DSPE
NH2
h
8.0
5.0
0.0
AA-PEG-hyd-DSPE
a
b
c
d
f
g
h
a
h
f
10.0
5.0
0.0
ppm

Supplementary figure 3 The ^{1}H NMR spectrum (dissolved in $CHCl_3$) of the DSPE-hyd-PEG-AA.
